# Nickel and low CO_2_-controlled motility in *Chlamydomonas *through complementation of a paralyzed flagella mutant with chemically regulated promoters

**DOI:** 10.1186/1471-2229-11-22

**Published:** 2011-01-25

**Authors:** Paola Ferrante, Dennis R Diener, Joel L Rosenbaum, Giovanni Giuliano

**Affiliations:** 1 ENEA, Casaccia Research Center, Via Anguillarese 301, 00123 Rome, Italy; 2 Department of Molecular, Cellular and Developmental Biology, Yale University, 06511 New Haven, CT, USA

## Abstract

**Background:**

*Chlamydomonas reinhardtii *is a model system for the biology of unicellular green algae. Chemically regulated promoters, such as the nickel-inducible *CYC6 *or the low CO_2_-inducible *CAH1 *promoter, may prove useful for expressing, at precise times during its cell cycle, proteins with relevant biological functions, or complementing mutants in genes encoding such proteins. To this date, this has not been reported for the above promoters.

**Results:**

We fused the *CYC6 *and *CAH1 *promoters to an HA-tagged *RSP3 *gene, encoding a protein of the flagellar radial spoke complex. The constructs were used for chemically regulated complementation of the *pf14 *mutant, carrying an ochre mutation in the *RSP3 *gene. 7 to 8% of the transformants showed cells with restored motility after induction with nickel or transfer to low CO_2 _conditions, but not in non-inducing conditions. Maximum complementation (5% motile cells) was reached with very different kinetics (5-6 hours for *CAH1*, 48 hours for *CYC6*). The two inducible promoters drive much lower levels of RSP3 protein expression than the constitutive *PSAD *promoter, which shows almost complete rescue of motility.

**Conclusions:**

To our knowledge, this is the first example of the use of the *CYC6 *or *CAH1 *promoters to perform a chemically regulated complementation of a *Chlamydomonas *mutant. Based on our data, the *CYC6 *and *CAH1 *promoters should be capable of fully complementing mutants in genes whose products exert their biological activity at low concentrations.

## Background

*Chlamydomonas reinhardtii *is a unicellular green alga, capable of both photosynthetic and fermentative growth. A plethora of mutants in relevant biological processes are available, and nuclear and chloroplast transformation are easy to perform [1]. Its 120-megabase genome has been completely sequenced [2]. *Chlamydomonas *combines functions typical of higher plants, such as the presence of a chloroplast endowed with two photosystems [3], of protozoa, such as the presence of motile flagella for swimming [4], and of archaea, such as the presence of sensory rhodopsins mediating phototaxis [5].

Flagellar motility in *Chlamydomonas *is dependent on dynein motors, which drive microtubule sliding, and a multitude of accessory proteins that control dynein activity, including radial spokes and the central pair complex. Immotile mutants missing individual subunits of these components have been identified and, in many cases, rescued by introducing the corresponding wild-type gene driven by its native promoter [6,7]. The first case of such complementation was achieved in a mutant, *pf14*, which has paralyzed flagella due to a premature stop codon in the gene encoding radial spoke protein 3 (RSP3) [8]. *RSP3 *encodes a protein mediating the anchoring to the axoneme of a cAMP-dependent protein kinase that regulates axonemal motility and dynein activity [9,10]. Flagellar motility can be restored by transformation of the mutant with the wild-type *RSP3 *gene [6], thus providing a nice biological assay for activity of the promoter driving *RSP3 *transcription.

Several chemically regulated promoters have been described in *Chlamydomonas*: the Nitrate Reductase (*NIT1*) promoter, induced by ammonium starvation [11]; the Carbonic Anhydrase (*CAH1*) promoter, induced by low CO_2 _[12]; and the Cytochrome C6 (*CYC6*) promoter, induced by copper (Cu) depletion or nickel (Ni) addition [13,14]. In all three cases, inducible expression has been demonstrated using reporter genes such as arylsulfatase or luciferase and, in the case of the *NIT1 *promoter, through complementation of a paralyzed flagellar mutant, *pf14*, by expressing the wild type form of the *RSP3 *gene [15]. No data are available, to our knowledge, on the capacity of the *CAH1 *and *CYC6 *inducible promoters to drive complementation of *Chlamydomonas *mutants.

To assess the capacity of the *CYC6 *and *CAH1 *promoters to complement the *pf14 *mutation in a chemically regulated fashion, we transformed the paralyzed *pf14 *mutant with the *RSP3 *gene under the control of the above-mentioned promoters and scored the swimming phenotype. The strong constitutive *PSAD *promoter [16] was used as a control.

## Results

### Constructs used for chemically inducible complementation

**Figure 1:**
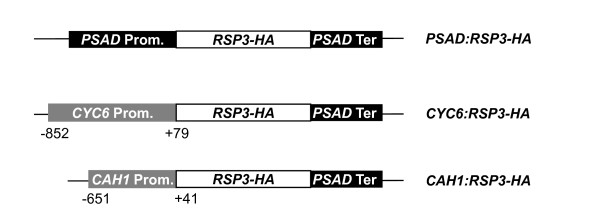
**Schematic maps of the constructs used**. The *RSP3 *sequence includes introns and is translationally fused to an HA tag. For details, see Methods.

The complete *RSP3 *gene (including introns) was translationally fused to a 9-amino acid HA epitope at its 3' end, to facilitate the immunodetection of the expressed protein [17]. The *RSP3-HA *hybrid gene was placed under the control of the *CYC6 *and *CAH1 *promoters, induced, respectively, by Ni and low CO_2 _[13,14,12] and, as a control, of the strong constitutive *PSAD *promoter [16]. The constructs are schematically represented in Figure 1.

### Constitutive complementation of the *pf14 *mutant driven by the *PSAD *promoter

**Figure 2:**
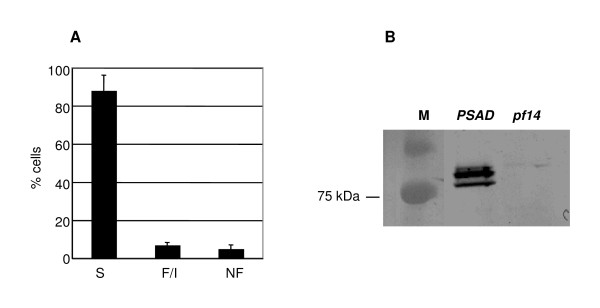
**Constitutive complementation of the *pf14 *mutant by the *PSAD:RSP3-HA *construct**. Panel A: Percentage of swimming (S), flagellated-immotile (F/I) and non-flagellated (NF) cells in a single, rescued transformant. Panel B: Western blotting of the *PSAD:RSP3-HA *transformant and *pf14 *mutant probed with the anti-HA antibody. M, molecular weight marker. Cells were grown in 24-well microtiter plates. For details, see Methods.

The *pf14 *mutant strain was transformed with the *PSAD:RSP3-HA *plasmid and 68 paromomycin-resistant transformants were grown for 4 hours without shaking in the light. Upon microscopic examination, about 40% of the transformants showed swimming cells. The average percentage of swimming cells was about 80% (Table 1). This result shows that the RSP3-HA fusion protein is able to rescue the *pf14 *mutant. The data of a representative transformant are shown in Figure 2. The majority of the cells (88%) were flagellated and motile (Panel A) and strong signals corresponding to the unphosphorylated (lower band) and phosphorylated (upper band) forms of the RSP3-HA protein were detected in a Western blot using the anti-HA antibody (Panel B).

**Table 1:** Percentage of rescued transformants showing swimming, and percentage of flagellated and swimming cells in the rescued transformants.

Construct	% rescued transformants	% flagellated cells in rescued transformants	% swimming cells in rescued transformants
*PSAD: RSP3-HA*	40	90 ± 10	80 ± 16
*CYC6: RSP3-HA*	8	12 ± 2	5 ± 0.8
*CAH1:RSP3-HA*	7	90 ± 10	5 ± 1.0

In this experiment 68 transformants were analyzed for each construct.

### Chemically inducible complementation of *pf14 *driven by the *CYC6 *and *CAH1 *promoters

*pf14 *cells were transformed with the *CYC6:RSP3-HA *and the *CAH1:RSP3-HA *plasmids and 68 transformed colonies were analyzed for each construct. Before analysis, the *CYC6:RSP3-HA *transformants were inoculated in TAP ENEA2 medium, allowing optimal expression of the *CYC6 *promoter, and expression was induced in the mid-log phase (6 × 10^6^-8 × 10^6 ^cells/ml) by adding 25 μM Ni [14]. We used a rather low Ni concentration, since higher concentrations cause detachment of flagella, preventing the scoring of the swimming phenotype (data not shown). The swimming phenotype was scored 48 hours after induction, when *CYC6 *promoter expression is maximal [14]. Approximately 8% of the transformants displayed swimming (Table 1) and, in these transformants, an average of 5% of the cells were motile. This difference with respect to the *PSAD:RSP3-HA *transformants is due to two factors: a much lower percentage of cells are flagellated in the *CYC6:RSP3-HA *transformants (12% *vs *90%) and, of these, a lower percentage are swimming (Table 1). As discussed below, we attribute this difference to a threshold effect. Movies of *PSAD:RSP3-HA *and *CYC6:RSP3-HA *transformants are available as Additional files 1 and 2.

In order to prevent loss of flagella at high cell densities (see below), cells were also induced with 25 μM Ni in early log phase (1 × 10^6^-2 × 10^6 ^cells/ml), but no rescue was observed (data not shown). This is consistent with the observation of Quinn et al. [13] that activation of the *CYC6 *promoter is stronger when the cells are induced at mid-late log phase, probably because Ni uptake is higher.

The *CAH1:RSP3-HA *transformants were grown in air containing 5% CO_2_, in minimal medium supplemented with extra phosphate buffer to keep the pH stable. Expression of the *CAH1 *promoter was induced in early-log phase by transferring the plate to air and cells were scored for swimming 6 hours after induction, when the *CAH1 *promoter shows high expression [12]. Approximately 7% of the transformants showed swimming and, as for the *CYC6:RSP3-HA *transformants, approximately 5% of the cells were motile in the rescued transformants (Table 1).

**Figure 3:**
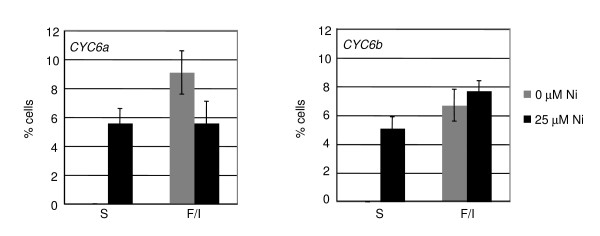
**Chemically inducible complementation of the *pf14 *mutant by the *CYC6:RSP3-HA *construct**. Percentage of swimming (S) and flagellated-immotile (F/I) cells of two transformants, 48 hours after Ni addition. The transformants were grown in TAP ENEA2 medium in 24-well microtiter plates and induced at mid-log phase with 25 μM Ni. For details, see Methods.

The percentage of swimming and flagellated-immotile cells was determined for two representative *CYC6:RSP3-HA *transformants showing restored motility, 48 hours after Ni addition (Figure 3), when the *CYC6 *promoter shows high expression [14] and cell density is high (1 × 10^7^-2 × 10^7 ^cells/ml). The percentage of swimming cells was about 5% in both cases, whereas the flagellated/immotile cells ranged between 5% and 8%. Loss of flagella is independent of addition of Ni at 25 μM, since it is observed also in the non-induced transformants at high cell densities (Figure 3, gray bars). Cell density-dependent loss of flagella is not observed in wild type or *PSAD:RSP3-HA *transformants, suggesting that continuous, or high level, expression of RSP3 prevents this phenomenon.

**Figure 4:**
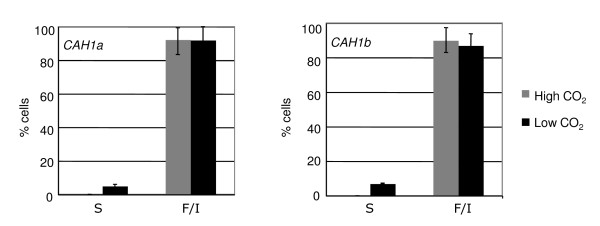
**Chemically inducible complementation of the *pf14 *mutant by the *CAH1:RSP3-HA *construct**. Percentage of swimming (S) and flagellated-immotile (F/I) cells of two transformants, 6 hours after induction by low CO_2_. The transformants were grown in minimal medium with extra phosphate in 24-well microtiter plates, under air containing 5% CO_2_, and induced at early log phase by shifting to air with no CO_2 _supplementation. For details, see Methods.

The percentage of swimming cells in two representative *CAH1:RSP3-HA *transformants was determined 6 hours after transfer to low CO_2 _(Figure 4), when the *CAH1 *promoter shows high expression [12]. In this case, cell density was low (2 × 10^6^-4 × 10^6 ^cells/ml) and the percentage of flagellated cells was high (approx. 90%). However, as for the *CYC6:RSP3-HA *transformants, the percentage of motile cells was low (5%-6% of total cells).

**Figure 5:**
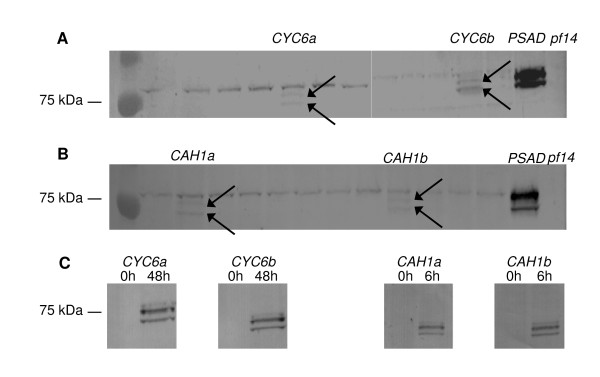
**Western blot of *CYC6:RSP3-HA *and *CAH1:RSP3-HA *transformants, probed with the anti-HA antibody**. Panels A and B: Screening of protein extracts *CYC6:RSP3-HA *and *CAH1:RSP3-HA *transformants, extracted, respectively, 48 h and 6 h after induction. Transformants that exhibit inducible swimming are labeled. Arrows point at the RPS3-HA bands. Cultures were grown and induced as in Figures 3 and 4, extracted, and 20 μg total proteins were loaded on each lane. Panel C: Re-analysis of transformants exhibiting inducible swimming (from Panels A and B). Cultures were grown and induced as in Figure 6, extracted, and 40 μg total proteins were loaded on each lane. For details, see Methods.

**Figure 6:**
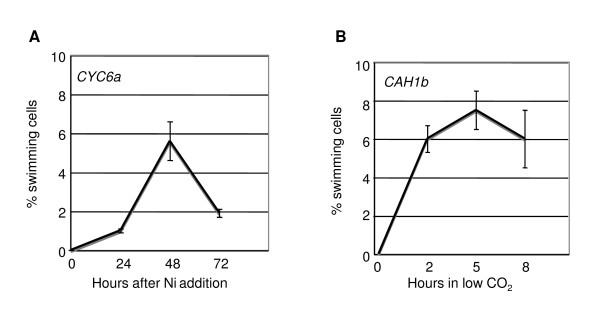
**Time course of inducible swimming in one *CYC6:RSP3-HA *(Panel A) and one *CAH1:RSP3-HA *(Panel B) transformant**. The *CYC6:RSP3-HA *transformant was grown in 6 ml in 6-well microtiter plates with shaking (120 rpm) and the *CAH1:RSP3-HA *transformant was grown in 150 ml in 250-ml Erlenmeyer flasks with bubbling.

Figure 5 (Panels A and B) shows a Western blot of several *CYC6:RSP3-HA *and *CAH1:RSP3-HA *transformants, grown in the same conditions of Figures 3 and 4, and probed with an anti-HA antibody. Only transformants showing motility in the swimming assay (Figures 3 and 4) showed the two bands corresponding to the RSP3 protein. The signal of the two bands is very weak compared to the *PSAD:RSP3-HA *transformants, suggesting that the low percentage of swimming cells is probably due to low expression of the RSP3 protein. The swimming transformants were re-grown in the same conditions used in Figure 6, and probed 0 h and 48 h (*CYC6 *transformants) or 0 h and 6 h (*CAH1 *transformants) after induction. The results (Panel C) show that the RSP3 protein is completely absent in non-induced, and readily detectable in induced cells.

### Kinetics of induction of the swimming phenotype

We then determined the kinetics of appearance of swimming cells in one representative *CYC6:RSP3-HA *and one *CAH1:RSP3-HA *transformant (Figure 6). In the case of the *CYC6 *promoter, swimming cells were observed as early as 24 hours after Ni addition. At 48 hours the number of swimming cells reached a maximum and then decreased at 72 hours. This is in agreement with the kinetics of activation of the *CYC6 *promoter, measured with the luciferase reporter gene, which reaches maximum activity after two days of induction and then decreases at three days [14]. In the *CAH1:RSP3-HA *transformant, swimming cells were observed as early as 2 hours after transfer to low CO_2_. The maximum number of swimming cells was reached 5 hours after transfer, and then declined at 8 hours. However, considering the standard deviations at 5 and 8 hours, this decline is not significant.

## Discussion

Through the use of the chemically regulated *CYC6 *and *CAH1 *promoters and of a genomic *RSP3 *clone fused to an HA epitope, we have achieved the chemically regulated motility of *Chlamydomonas *cells. While the vast majority of cells showed motility when *RSP3 *expression was driven by the constitutive *PSAD *promoter, only a minority (5%) of cells showed motility after induction of the *CYC6 *and *CAH1 *promoters. This is probably due to a threshold effect: the levels of RSP3-HA protein driven by *PSAD *are much higher than those driven by *CYC6 *and *CAH1*. The low levels of RSP3-HA protein expressed from the *CAH1 *promoter after 6 hours of induction contrast markedly with the high levels of CAH1 protein expressed from the endogenous gene (data not shown). Low expression of exogenously introduced constructs in *Chlamydomonas *is a well-known phenomenon, which has been attributed to gene silencing [18].

The low levels of RSP3-HA protein expressed from the *CYC6 *promoter after 48 hours of induction are also puzzling, since, in TAP ENEA2 medium, the *CYC6 *promoter is able to drive levels of luciferase expression comparable to those driven by *PSAD *[14]. We attribute this difference in RSP3 *vs *luciferase expression to the fact that RSP3 accumulates over time when it is expressed from *PSAD*, while expression for 48 hours (from *CYC6*) or 6 hours (from *CAH1*) allows accumulation of low RSP3 levels (Figure 5). This implies that the RSP3 protein is more stable than luciferase (whose estimated half-life in *Chlamydomonas *is <2 hours [19]). Whatever the case, the low levels of expressed RSP3-HA protein are sufficient for achieving motility in 5% of the transformed cells. To our knowledge, this is the first example of the use of the *CYC6 *and *CAH1 *promoters for achieving chemically regulated complementation of a *Chlamydomonas *mutant, as well as the first example of metal- or CO_2_-regulated motility engineered in a living organism. The partial complementation observed is probably due to the fact that the RSP3 protein, to exert its function, is required in high concentrations. Although the number of radial spokes required to restore motility to flagella is not known, each wild type flagellum contains approximately 2,000 radial spokes [20].

Zhang and Lefebvre [15] have used the *RSP3 *gene under the control of the ammonium-repressible *NIT1 *promoter to complement the *pf14 *mutant in a nitrogen source-dependent fashion. In that study, 81 out of 2,000 cotransformants showed motility in permissive conditions, i.e. a fraction of about 4%, comparable to the 7-8% reported here for the *CYC6 *and *CAH1 *promoters. At least one of the transformants, containing multiple copies of the *NIT1:RSP3 *plasmid, showed full complementation, i.e. a large number of swimming cells, a fact we did not encounter in the case of the *CYC6 *and *CAH1 *promoters, probably due to the smaller number of colonies screened in our study and to the fact that the vast majority of the insertions, in our case, are single-copy (Additional file 3). Whatever the case, the frequency of swimming transformants obtained with the strong *PSAD *promoter is 40% (Table 1), i.e. much higher than what can be obtained using either the *NIT1*, *CYC6*, or *CAH1 *promoters in permissive conditions. A chemically regulated promoter system allowing such high complementation efficiencies in permissive conditions has yet to be worked out.

## Conclusions

We have demonstrated low level, chemically regulated complementation of the paralyzed flagella *pf14 *mutant by the *RSP3 *gene, encoding a component of the flagellar radial spoke complex, cloned under the control of the *CYC6 *and *CAH1 *promoters. Maximum complementation is reached with very different kinetics (6 hours for *CAH1*, 48 hours for *CYC6*). In principle, these promoters should be capable of fully complementing mutants in genes whose products exert their biological activity at low concentrations (e.g. receptor/signalling protein kinases). Test of this hypothesis is under way, as well as the optimization of the *CYC6 *and *CAH1 *promoters, for full complementation of mutants in genes encoding abundant intracellular proteins.

## Methods

### Strains and culture conditions

The paralyzed flagella mutant *pf14 *[8] was used for all experiments. Nuclear transformation was performed as described [21]. Plasmids were digested with Sca I and 300 ng of DNA were used for each transformation. Transformants were selected on TAP agar plates containing paromomycin (10 μg/ml).

Unless indicated differently, cells were grown photomixotrophically in TAP medium at 25°C under irradiation (16 L: 8 D) with fluorescent white light (200 μE m^-2 ^s^-1^). For the initial screening, 68 transformants for each construct were grown in 200 μL in 96-well microtiter plates with shaking (900 rpm). For quantitative measurements of motility (Figures 2, 3, 4, 5A and 5B), transformants were grown in 2 mL in 24-well microtiter plates with shaking (500 rpm). For the experiments described in Figure 5C and in Figure 6 cells were grown in 6 ml in 6-well microtiter plates with shaking (120 rpm) (*CYC6 *transformants) or in 150 ml in 250-ml Erlenmeyer flasks (*CAH1 *transformants) with bubbling. The plates were covered with Breathe-Easy membrane (Diversified Biotech, cat. BEM-1), to prevent evaporation without limiting gas and light exchange. For Ni induction, cells were grown in TAP ENEA2 medium [14] and induced at mid-log phase (6 × 10^6^-8 × 10^6 ^cells/ml) by adding 25 μM Ni. For low-CO_2 _induction, cells were grown in minimal medium with doubled phosphate buffer concentration, to keep the pH stable in high CO_2 _conditions [22], in air containing 5% CO_2 _and induced by shifting to air in early log phase (1 × 10^6^-2 × 10^6 ^cells/ml).

### Plasmid construction

The complete *RSP3 *gene (including introns) was amplified using the following oligonucleotides:

Forward: GC**TCTAGA**ATGGTGCAGGCTAAGGCGCAGC

Reverse: GA**AGATCT**TTA*GGCGTAGTCGGGCACGTCGTAGGGGTA*CGCGCCCTCCGCCTCGGCGAAC

The forward oligonucleotide inserts an Xba I restriction site, the reverse oligonucleotide inserts a 9- amino acid HA-tag (the corresponding nucleotide sequence is in italics) followed by a TAA stop codon and a Bgl II restriction site (both restriction sites are in bold). The two oligonucleotides were used to amplify the *RSP3 *gene and the *RSP3-HA *insert was used to replace the *cRLuc *sequence in the *PSAD:cRLuc *and *CYC6:cRLuc *plasmids [14]. To construct the *CAH1: RSP3-HA *plasmid, the -651 +41 region of the *CAH1 *promoter was amplified with the following oligonucleotides: *CAH1 *forward: CCG**CTCGAG**TCAGCTTCTCTCCCGCCAGC*; CAH1 *reverse: GC**TCTAGA**GGTGTTCAAGTGGGTTGCAG. The *CAH1 *forward oligonucleotide inserts an Xho I restriction site, the *CAH1 *reverse primers inserts an Xba I restriction site (both restriction sites are in bold). The two oligonucleotides were used to amplify the -651 +41 region of the *CAH1 *promoter and the insert obtained was used to replace the *CYC6 *sequence in the *CYC6:RSP3-HA *plasmid.

Transgene copy number was estimated *via *quantitative Real-Time PCR. Total DNA was extracted from the *PSAD:RSP3-HA*, *CYC6-RSP3-HA *and *CAH1-RSP3-HA *transformants and from the *pf14 *strain using the DNeasy Plant Mini Kit (Qiagen, cat. N. 69104). A calibration curve (Additional file 3) was made by mixing 10 ng of total DNA from the *pf14 *strain with different amounts of linearized *PSAD:RSP3-HA *plasmid, corresponding to 0.5, 1 and 2 copies per genome. The *CYC6 *gene was used as an internal standard for normalization. The oligonucleotides used to amplify the *RSP3-HA *transgene are: *RSP3HA *forward: TACGCCTAAAGATCTGAATTCGG; *RSP3HA *reverse: TCAGCGAAATCGGCCATC. These oligonucleotides amplify the *PSAD:RSP3-HA*, *CYC6:RSP3-HA*, *CAH1:RSP3-HA *constructs and the corresponding transformants, but not the endogenous *RSP3 *gene. The oligonucleotides used to amplify the *CYC6 *gene are: *CYC6 *forward; TGGATTTCCTCCTCCGACAG; *CYC6 *reverse: CTGGATGGCGGCTTCAAG. The Real-Time PCR amplification conditions were: 95°C, 10 min; 50 cycles of 95°C, 15 sec and 60°C, 1 min. The SYBR Green PCR Master Mix (Applied, Cat. N. 4309155) was used in a final volume of 20 μl.

### Proteins electrophoresis and Western blotting

Two ml of *Chlamydomonas *culture were centrifuged at 10,000 × g at room temperature for 1 minute and the cell pellets were resuspended in 300 μl of 60 mM DTT, 60 mM Na_2_CO_3_, 2% SDS, 12% sucrose and shaken for 20 minutes at room temperature to extract the proteins. The protein extracts were centrifuged at 10,000 × g for 1 minute and the supernatant collected. To measure protein concentration, 10 μl of protein extracts were mixed with 800 μl of 0.5% Amido Black in 90% methanol and 10% glacial acetic acid. The samples were vortexed and centrifuged at 10,000 × g at 4°C for 10 minutes. The pellets were washed two times with 90% methanol and 10% glacial acetic acid at 4°C. Finally the pellets were resuspended in 800 μl of 0.2 M NaOH and the absorbance measured at 615 nm. To calculate protein concentration a standard curve with BSA was used.

Western blot analyses were performed on total protein extracts obtained as described above. About 30 μg protein/sample were separated on an 8% SDS-PAGE gel [23]. Proteins were blotted on nitrocellulose membrane in a buffer containing 25 mM Tris, 192 mM glycine, 20% ethanol for 2 hours at 250 mA using a Hoefer TE22 apparatus. A commercial anti-HA antibody (Ascites Fluid Mono HA 11, 16B12, Covance) was used in a 1:250 dilution. The secondary antibody (anti-mouse, phosphatase conjugated, Thermo Scientific 31325) was used in a 1:2500 dilution. Detection was performed placing the nitrocellulose membrane in 100 mM NaCl, 5 mM MgCl_2_, 100 mM Tris (pH 9.5) containing Nitrotetrazolium Blue chloride (NBT) 0.33 mg/ml and 5-Bromo-4-chloro-3-indolyl phosphate disodium salt (BCIP) 0.165 mg/ml. To stop the reaction, the membrane was rinsed with Phosphate-Buffered saline (PBS) containing 20 mM EDTA.

### Microscopy

Cell motility and the presence of flagella were assessed using an Olympus BX41 microscope with 16 X and 40 X objective lenses, respectively. Movies were recorded with a Cool Snap HQ camera (Photometrics) on a Nikon Eclipse TE2000 inverted microscope using a 10 X objective lens.

## Authors' contributions

PF performed experiments. DRD and GG supervised experiments. All authors designed experiments, interpreted data and wrote the manuscript.

## Supplementary Material

Additional file 1 **Motility of a *PSAD:RSP3-HA *transformant**. Movie showing the motility of a *PSAD:RSP3-HA *transformant. Click here for file

Additional file 2 **Motility of a *CYC6:RSP3-HA *transformant, 48 hours after Ni induction**. Movie showing the motility of a *CYC6:RSP3-HA *transformant, 48 hours after Ni induction. Click here for file

Additional file 3 **Estimation of transgene copy number by quantitative Real-Time PCR**. Figure showing the estimation of transgene copy number by Real Time PCR. Click here for file
