# Supplementary material for: Nickel and low CO2-controlled motility in Chlamydomonas through complementation of a paralyzed flagella mutant with chemically regulated promoters
Source: BMC Plant Biol. 2011 Jan 25;11:22. doi: 10.1186/1471-2229-11-22 (PMC3038898; doi:10.1186/1471-2229-11-22)
Supplement: Additional file 3 — Estimation of transgene copy number by quantitative Real-Time PCR. Figure showing the estimation of transgene copy number by Real Time PCR. [file 1471-2229-11-22-S3.PDF]

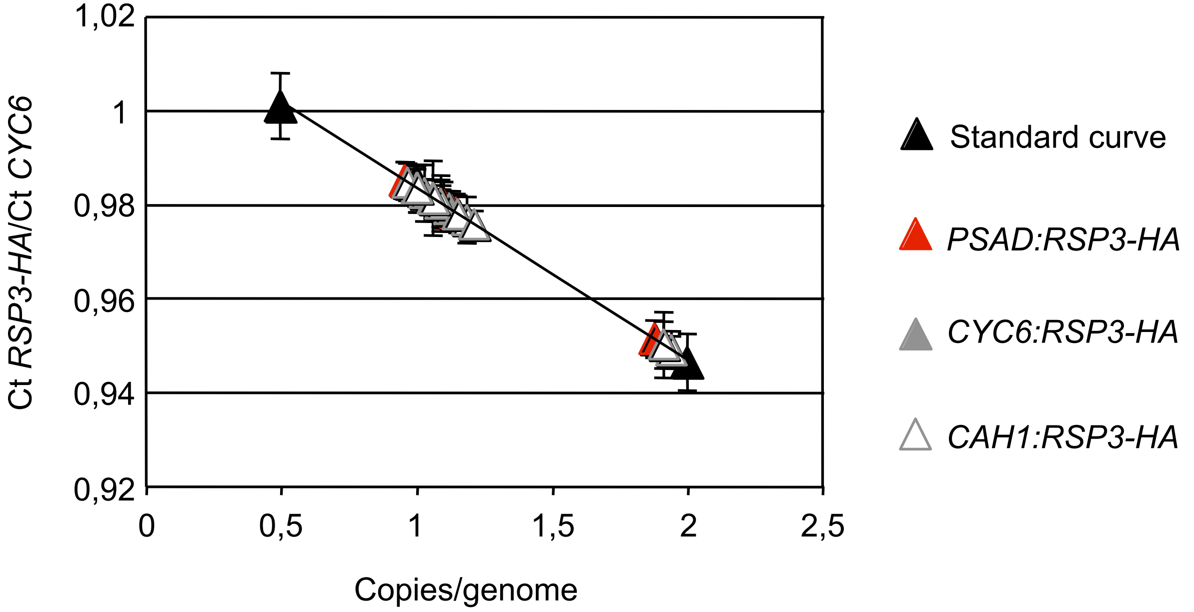

Additional File 3 - Estimation of transgene copy number by quantitative Real-Time PCR.
The standard curve was made using different amounts of the *PSAD:RSP3-HA* construct corresponding to 0.5, 1 and 2 copies per genome mixed to 10 ng of genomic DNA extracted from *pf14* cells. The ratio between the threshold cycles (Ct) of the *RSP3HA* and *CYC6* (internal standard) amplicons is plotted on the y axis, the number of copies on the x axis. The R^2 of the standard curve is 0.9979. The Ct ratios of ten different *PSAD:RSP3-HA*, ten *CYC6:RSP3-HA* and ten *CAH1:RSP3-HA* transformants are shown. The majority of the transformants carry single copies of the transgenes, with only 2 *PSAD:RSP3-HA*, 1 *CYC6:RSP3-HA*, and 2 *CAH1:RSP3-HA* carrying two copies. All the transformants showing expression of the transgene are single-copy. For details, see Methods.
